# Equity in health personnel financing after Universal Coverage: evidence from Thai Ministry of Public Health’s hospitals from 2008–2012

**DOI:** 10.1186/s12960-015-0046-y

**Published:** 2015-07-18

**Authors:** Wilailuk Ruangratanatrai, Somrat Lertmaharit, Piya Hanvoravongchai

**Affiliations:** Bureau of Policy and Strategy, the Office of the Permanent Secretary, Ministry of Public Health, Bangkok, Thailand; Department of Preventive and Social Medicine, Faculty of Medicine, Chulalongkorn University, Bangkok, Thailand; Thailand Research Center on Health Services System, Faculty of Medicine, Chulalongkorn University, Bangkok, Thailand

**Keywords:** Equity, Health personnel, Health financing, Compensation, Thailand

## Abstract

**Background:**

Shortage and maldistribution of the health workforce is a major problem in the Thai health system. The expansion of healthcare access to achieve universal health coverage placed additional demand on the health system especially on the health workers in the public sector who are the major providers of health services. At the same time, the reform in hospital payment methods resulted in a lower share of funding from the government budgetary system and higher share of revenue from health insurance. This allowed public hospitals more flexibility in hiring additional staff. Financial measures and incentives such as special allowances for non-private practice and additional payments for remote staff have been implemented to attract and retain them. To understand the distributional effect of such change in health workforce financing, this study evaluates the equity in health workforce financing for 838 hospitals under the Ministry of Public Health across all 75 provinces from 2008–2012.

**Methods:**

Data were collected from routine reports of public hospital financing from the Ministry of Public Health with specific identification on health workforce spending. The components and sources of health workforce financing were descriptively analysed based on the geographic location of the hospitals, their size and the core hospital functions. Inequalities in health workforce financing across provinces were assessed. We calculated the Gini coefficient and concentration index to explore horizontal and vertical inequity in the public sector health workforce financing in Thailand. Separate analyses were carried out for funding from government budget and funding from hospital revenue to understand the difference between the two financial sources.

**Results:**

Health workforce financing accounted for about half of all hospital non-capital expenses in 2012, about a 30 % increase from the level of spending in 2008. Almost one third of the workforce financing came from hospital revenue, an increase from only one fourth 5 years earlier. The study reveals a big difference in health workforce expenditure *per capita* across provinces. Health workforce spending from government budget was less equal than that from hospital revenues as shown by the higher Gini coefficient. The concentration indices show that the financing of hospital workforce was higher *per capita* in lower resource provinces.

**Conclusion:**

Our analysis of equalities in health workforce spending shows an improving trend in equity across provinces from 2008–2012. Expansion of healthcare and health insurance coverage and financing reform towards a demand-side financing helped improve the distribution of funding for health workforce across the provinces. The findings from this study can be useful for other countries with ongoing reform towards universal health coverage.

## Background

Shortage and maldistribution of the health workforce is one of the major problems of the health systems in many ASEAN countries [1]. The average density of the health workforce as measured by the number of doctors, nurses and midwives combined per 1000 population in ASEAN countries is still lower than global average. At the national level, there are critical shortages in five countries: Cambodia, Indonesia Laos, Myanmar and Vietnam.

In Thailand, even though the workforce density of health workers is not below the critical shortage boundary, there is maldistribution of the health workforce with shortages in underserved rural areas [1]. For example, the ratio of doctors and the ratio of nurses *per capita* in Bangkok are, respectively, 2.4 and 1.7 times the density of the central region, while the densities in the northeast region are only 50 % or 60 % of the densities of the central region [2]. Similar to other countries, it is difficult to attract and retain human resources for health (HRH) to work in rural areas in Thailand.

A number of policies have been implemented with the aim to retain key health professionals in the public sector. The examples are the expansion of medical doctor training capacity through various publicly supported projects such as the training for doctors to be positioned in the three southernmost provinces of Thailand and one scholarship per subdistrict to recruit students to study medicine or nursing and return to work in their home town. Despite these measures, there are still difficulties retaining staff in the public sector especially in remote areas. A number of financial measures and incentives such as special allowances for non-private practice and additional payments for remote staff have also been implemented. However, there has been no study to assess the equity in HRH financing in the public sector in Thailand.

Equity is a normative concept that is not the same as equality. It relies on social perception of what is considered as fair or moral [3-6]. It requires adequate consideration of two key dimensions of equity namely horizontal equity and vertical equity. Horizontal equity is the equal treatment of the equals while vertical equity takes into account the variation of population characteristics into defining what would be fair. Measurement of equality therefore only satisfies the assessment of horizontal equity but not vertical equity.

There are many ways to measure inequality such as a basic comparison between the ratios of the highest to the lowest. This approach is easy to measure and easy to understand but may not be able to capture adequately the differences in between. Many composite indices also exist to summarize the inequalities such as the Gini coefficients, Theil index, Atkinson index and standard deviation of the logs [7]. However, these summary indices do not measure vertical inequity which requires a different set of summary indicators. For example, the Kakwani index, which was invented to assess the progressivity of the tax system, was also used to measure the vertical inequality of healthcare financing [8].

Equity analyses have been employed in the health sector including for the analysis of health workforce distribution [9-13]. Previous studies on equity of human resource for health in Thailand frequently use the population ratio of health personnel to compare the difference in the distribution of human resource for health [14, 15]. However, most studies did not provide a summary measure to help demonstrate changes over time of the equity situation. Additionally, they only assess workforce densities but did not look into the differences in health worker financing which may provide a more relevant picture of health service systems for policy interventions.

Thailand has 76 provinces in addition to Bangkok. Each province has a population size varying from 181 181 to 2,475,598 persons. At the national level, the health service system consists of public and private hospitals. The Ministry of Public Health (MoPH) is the main agency that provides health services in the provinces while private hospitals concentrate in Bangkok and big cities. Almost all of the MoPH hospitals are mainly under the management of the MoPH’s Office of Permanent Secretary (OPS) and can be classified as regional hospitals, general hospitals and community hospitals. In the year 2012, MoPH OPS has 836 hospitals with inpatient services; most of them were district-level (community) hospitals (89 %), and the remaining are general hospitals (8 %) and regional hospitals (3 %) [16]. MoPH OPS hospitals are the main inpatient service providers in the country and constitute 68 % of all hospitals or 65 % of all public and private hospital beds in Thailand in 2012 [16]. These hospitals employed over a hundred thousand health professional staff including 10 730 doctors and 73 692 nurses. They are approximately two fifths and three fifths of the total doctors and nurses in the whole country, respectively.

Main funding sources for MoPH OPS hospitals came from government budget and hospital revenue. The government budget for these hospitals was mainly for compensation and benefits of civil servants and permanent employees who worked as hospital staff, as well as for hospital operating costs (excluding medical care). Hospitals also got revenues for the medical care service they provided from health insurance schemes or from patients’ out-of-pocket payments. The financial reform towards universal health coverage in Thailand, which started in 2002, led to a major change in the population coverage of health insurance and hospital payment mechanisms [17]. Hospitals received predominantly capitation payments for outpatient services based on the number of population registered with them and case-based payments for inpatient services they provided. Since 2002, the proportion of hospital revenues from health insurance increased while the proportion of funding from government budget decreased.

The objective of this study is to evaluate health workforce financing in public hospitals across all provinces in Thailand by assessing inequity in the distribution of these resources. We excluded the capital city, Bangkok, due to its special nature as a national referral centre and the limited role of public sector providers in the province. We only considered the financing of public MoPH OPS hospitals.

## Methods

Data for HRH financing were collected from routine reports of MoPH OPS hospital financing in the fiscal year 2008–2012. The data were collected from all 838 MoPH OPS hospitals in the country. Financial data prior to 2008 were less reliable and of low quality so they were omitted.

The datasets contain information on actual expenses by each hospital and include financing for HRH-related expenses as well as other non-HRH expenses. The data on HRH expenses included salary, position-related payment, bonus payment, benefits, other financial allowances and other HRH-related payments. The data on non-HRH expenses were mainly for drugs and other medicines, medical supplies, office supplies and utilities such as electricity and water.

There were 11 to 18 hospitals with incomplete data in each of the study years. For these hospitals, missing values were replaced with average value of corresponding expenditure items from other hospitals of similar size (in terms of hospital beds) in the same year. Monthly data were aggregated into annual figures of each hospital and combined into province-level figures. The category of expenses and the source of funding were analysed to provide a descriptive picture of HRH financing across the provinces.

Inequalities in health workforce financing across provinces were assessed using a Gini-like coefficient. The Gini coefficient of inequality is a commonly used measure of income inequality [18]. It can be demonstrated using a Lorenz curve of income distribution, which plots the share of cumulative income enjoyed by the relative share of the population. Instead of income, we calculated the Gini coefficient of health workforce financing at the province level by using health workforce financing *per capita* as the variable of interest.

Additionally, inequality in health workforce financing across provinces in relation to provincial income was also assessed using the concentration index. It is a summary measure that is frequently used for inequity assessment across income gradients. This allows the income-related inequality of HRH financing to be quantified, and the relationship between the two can be presented as a concentration curve [19]. In this study, we used the gross provincial product *per capita* as an income-related measure for province ranking in the calculation.

Calculation of the Gini coefficient and concentration index for health workforce financing was done using the inequal command of the STATA statistical software Release 11 [20]. Separate analyses were carried out for funding from government budget and funding from hospital revenue to understand the difference between the two financial sources.

## Results

The results show that the overall level of hospital financing and HRH spending at the province level had been increasing every year. More than half of hospital financing was for health workforce, with the share in total expenses rising from 42 % in 2008 to 51 % in 2012. From 2008 to 2012, overall MoPH OPS hospital expenses increased by 9 %, with HRH-related expenses increased by 31 % while non-HRH operating expenses decreased by 8 %. The proportion of salary in HRH expenses decreased from 72 % to 67 % in 2012 while the proportion of other allowances and benefits increased continuously (Table 1).

**Table 1:** **Overall health workforce spending in the hospitals under the Ministry of Public Health in the fiscal year 2008–2012 (million USD)**

Year	HRH spending				Non-HRH spending	Total
	Salary/wages	Allowances	Benefits	Total		
2008	1457	483	91	2032	2787	4819
2009	1525	629	118	2269	3207	5476
2010	1588	773	116	2480	3499	5979
2011	1671	735	119	2525	2554	5079
2012	1784	774	113	2670	2575	5244

Note: in 2012 value, 1 USD = 31.5 Baht

More than half of HRH financing came from government budget. HRH financing from the government budget increased about 12 % over the 5-year period, while HRH financing from hospital revenues increased about 61 %. Consequently, the shares of the government budget in HRH financing slowly decreased over time from 62 % in 2008 to around 53 % in 2012 (Table 2).

**Table 2:** **Sources of financing in the hospitals under the Ministry of Public Health in the fiscal year 2008–2012 (million USD)**

Year	Government budget (%)	Hospital revenues (%)	Unspecified sources (%)	Total
2008	1265 (62.2 %)	541 (26.6 %)	227 (11.2 %)	2033
2009	1287 (56.7 %)	668 (29.4 %)	315 (13.9 %)	2270
2010	1314 (53.0 %)	765 (30.8 %)	401(16.2 %)	2480
2011	1353 (53.6 %)	807 (32.0 %)	364 (14.4 %)	2525
2012	1416 (53.0 %)	870 (32.5 %)	387 (14.5 %)	2673

### Analysis of inequality of HRH spending *per capita* across provinces

There were significant differences in health workforce expenditure *per capita* across provinces throughout the study period. Average health workforce spending *per capita* rose from 36.4 USD *per capita* in 2008 to 46.5 USD per person in 2012. The ratio between the average spending level in the province with the highest HRH spending *per capita* and the level in the province with the lowest spending level was 6.6 times in 2008. The gap reduced in 2012, but the ratio was still high at 5.6 times (Table 3).

**Table 3:** Health workforce expenditure *per capita* across provinces

Year	Health workforce expenditure *per capita* (USD)			Max vs min ratio
	Average	Min	Max	
2008	36.38	13.21	87.81	6.6
2009	40.38	15.71	89.84	5.7
2010	43.78	17.40	95.81	5.5
2011	44.35	16.95	93.90	5.5
2012	46.51	17.90	100.83	5.6

Note: in 2012 value, weighted by population size

**Fig. 1:**
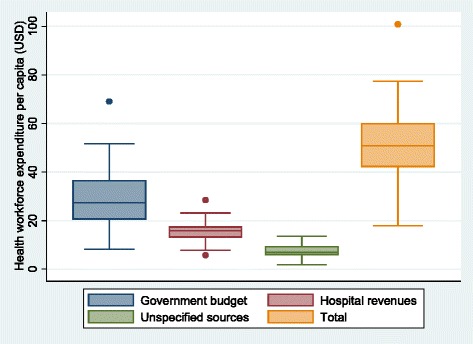
Health workforce expenditure per capita across provinces fiscal year 2012

**Fig. 2:**
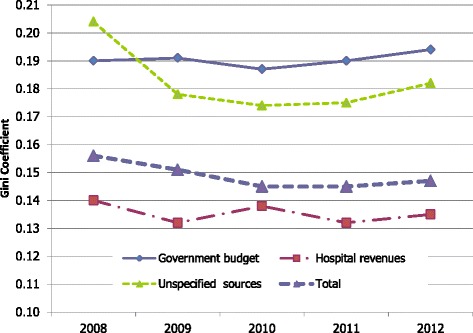
Gini coefficients of HRH spending *per capita* across provinces by source of funding

Looking at inequality in HRH financing per capita across provinces, financing from government budget was less equal than financing from hospital revenue. Figure 1 shows the box plot of the distribution of HRH spending *per capita* across provinces comparing different sources of financing in 2012. The historical trend of Gini coefficients for overall HRH financing and Gini coefficients by funding source is shown in Fig. 2.

**Fig. 3:**
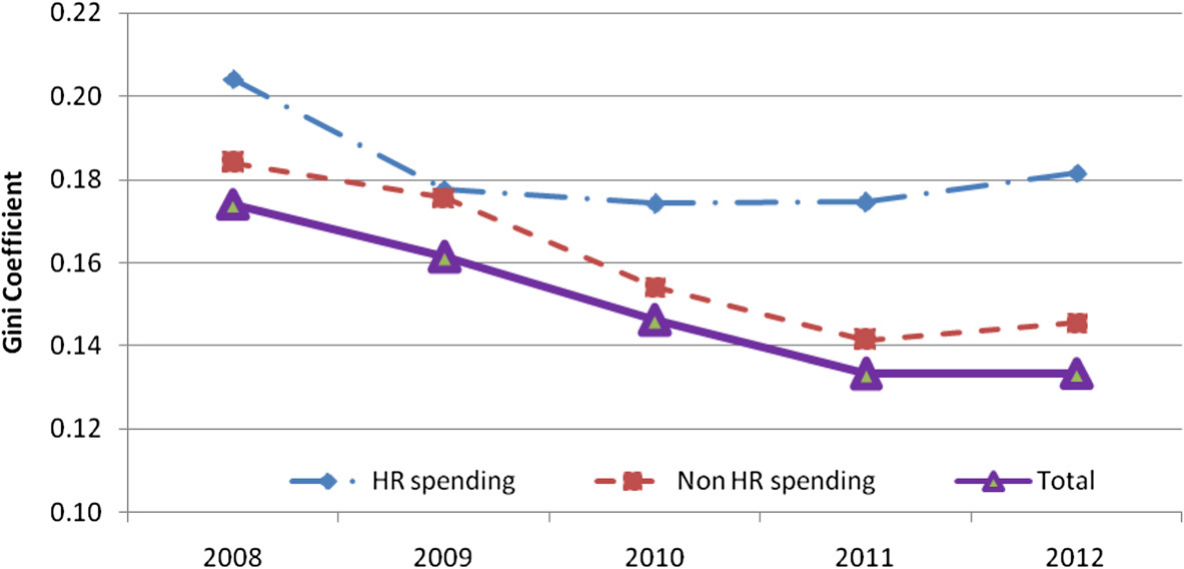
Gini coefficient of HRH spending *per capita* and non-HRH spending *per capita* across provinces

Figure 3 shows the trend in inequalities in HRH and non-HRH spending. Comparing HRH spending to non-HRH spending, we found that HRH spending *per capita* was less equally distributed than non-HRH spending in 2008. Moreover, inequality in non-HRH spending reduced over time while inequality in HRH spending did not.

### Analysis of inequality in HRH spending *per capita* in relation to provincial income

**Fig. 4:**
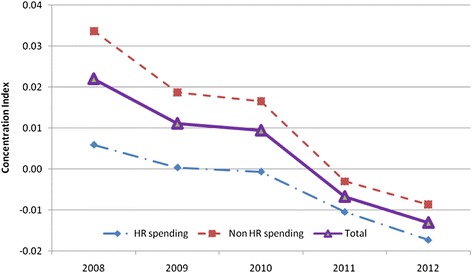
Concentration indices of HRH and non-HRH spending *per capita* across provinces over time

When ranked by provincial income level *per capita*, it was shown that both HRH and non-HRH funding sources were allocated more to the richer provinces in 2008. The HRH spending was, however, more pro-poor than the non-HRH spending as shown by the lower concentration index. Nevertheless, both funding sources became more equitable over time as shown by the lower CI over time even though the magnitude of change may be relatively small. Figure 4 shows the declining trend of CIs for all funding sources, with a bigger decline for non-HRH financing.

**Fig. 5:**
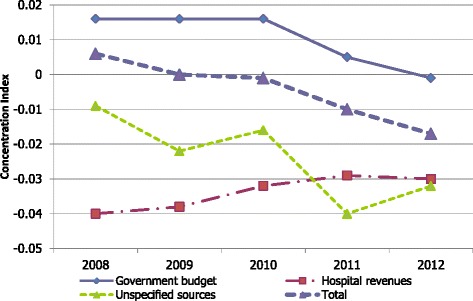
Concentration indices of different sources of HRH spending *per capita* across provinces over time

HRH spending *per capita* from government budget was higher for richer provinces as shown by the positive concentration indices in Fig. 5. Funding from hospitals’ own revenues was more equitable as shown by the negative concentration indices. The difference between the two funding sources decreased over time with HRH financing from government budget becoming more pro-poor while the HRH financing from hospital revenue were less pro-poor. However, these changes were relatively small as shown by the changes in the concentration index of less than 0.03.

## Discussion

In Thailand, HRH financing for MoPH OPS hospitals increased every year with the rate of increase even higher than the inflation rate. Two factors contributed to such a rapid rise in HRH spending. Firstly, the hospitals increased the number of their healthcare staff despite the restrictions on the size of civil servants following the central government’s zero growth policy for civil service workforce implemented since 2001 [21]. Nevertheless, these hospitals could still use their revenues to hire more staff, in order to cope with the rising demand for healthcare services. The effect of this decentralization of financial decision to allow temporary hiring using hospital revenues resulted in a bigger share of HRH spending from hospital revenues.

Secondly, there were high costs from the financial incentives provided under the government policies to attract and retain staff to work in the public sector especially in remote areas. The level of compensation could be very high especially for doctors and dentists; in many cases, up to twice the regular salary. For example, in 2008, MoPH started to provide additional allowances for health professionals working in a community hospital. The amount of allowance varied by profession, the duration of practice in that community and the remoteness of the hospital location. Doctors and dentists could receive up to 2222 USD per month from such an allowance. There were also other HRH performance payments, for example, for surgery and medical procedures performed after office hours.

The rising costs of health workforce financing in Thailand follow the global trend. As reported in a study by Hernandez et al. from WHO, funding for salaried health workers in 106 countries increased quicker than the increase in GDP [22]. There is a continuously increasing trend in the level of allowances for health personnel in several middle-income developing countries. One difference, however, is that in Thailand the share of health workforce spending in the overall hospital spending increased. This is different from the global trend presented in the above-mentioned study that shows that the health workforce share of total health spending decreased over time, possibly with more spending on technology or capital-intensive items [22]. This could be due to the role of the health financing system and the three major health insurance schemes that use highly cost conscious payment mechanisms to effectively control costs of care [23].

### Inequity in health workforce financing across provinces

Our analysis shows that there is a huge discrepancy in health workforce financing across provinces. The ratio of HRH spending *per capita* from the highest spending province to the lowest spending province was as high as over six times in 2008. Such difference could be the result of a historical pattern in HRH deployment where staff are allocated to hospitals according to their size. With hospital beds more concentrated in bigger provinces, more civil service posts were assigned to those richer provinces.

The government budget was mainly allocated for civil servant salaries and benefits. The civil service posts mostly follow the infrastructure distribution such as hospital size or number of beds. This means bigger hospitals in richer provinces tend to get more civil service posts historically. The civil service workforce also hardly increased over time due to the government’s policy to restrict the growth of the workforce. Existing civil servants working in the hospitals mostly do not relocate to smaller or more remote hospitals. If they do, they tend to request for relocation to bigger hospitals or to richer provinces for career advancement or for family reasons. This generally resulted in no improvement or a worsening geographical distribution of HRH financing from the government budget.

The change in hospitals’ financing allocation methods following the universal health coverage reform in 2002 led to a continuous decline in the share of hospital operating costs from the government budget. Prior to UCS initiation in 2002, MoPH OPS hospitals relied mainly on government budget with a limited share of income coming from hospital revenues. After 2002, hospitals received more of their income from third-party payers such as the Universal Coverage Scheme (UCS) and Social Security Scheme (SSS) based on the number of beneficiaries they cover. Both the National Health Security Office (NHSO) who oversees UCS and the Social Security Office (SSO) who oversees SSS pay hospitals mainly by capitation rate for outpatient services and case-based payment with the global budget for inpatient care. This was a shift from supply-side financing (government budget based on hospital beds or number of existing staff) to demand-side financing (population size and number of cases) which means hospitals in the region with a higher population density got a chance to receive a higher amount of funding because they serve more people.

With the increasing share of hospital financing from hospital revenues due to an increase in population coverage and the change in payment mechanism, hospitals have more flexibility in their use of funds and hence in their health workforce financing decisions. The hospital revenues can be used with much fewer restrictions compared to the centralized civil service system. Hospitals in areas of need can hire more temporary employees to respond to their staff shortages. This contributed to a better distribution of health workforce financing. As a result, an increase in health professional density per population was found in the northeast region where there were more shortages before UCS implementation in 2001.

Even though there was some improvement in inequality in health workforce financing over time, it is of concern that the inequality in the government budget did not improve enough and the overall HRH inequality trend became stagnant in the last three years. One major limitation in the progress is the reluctance of the Ministry of Public Health to move their civil service staff from higher density areas to lower density remote areas. It is also common for senior civil service staff to request to move to bigger or more developed provinces making the situation worse. It is important that policy makers and key stakeholders be aware of the inequality patterns in health workforce financing and develop relevant policies to continue the progress. Strategies and policies for rural recruitment and retention as well as reallocation of civil service staff positions especially the assignment of new staff posts to remote areas or provinces with relatively lower existing staff density should be emphasized in the MoPH HRH policy.

This study has a number of limitations. First, it only covers public hospitals under MoPH OPS. Private hospitals or clinics were not included in the study, so the analysis of inequality could not be representative of the situation of health workforce financing of the whole country. Nevertheless, the public financing component could be considered more important given its mandate to improve access and equity, so the analysis of only MoPH OPS hospitals should be satisfactory. Second, the analysis of the health workforce financing distribution was based on health workforce financing *per capita*. The number of population in the province (*capita*) may reflect health needs in the area, but it may not be adequate if there are large discrepancies across provinces such as in terms of population demographics or epidemiology profile. Future study could include better adjustment of health needs in the analysis. Third, the analysis of health workforce financing cannot provide a complete picture of healthcare inequality as it only considers the supply side and did not take into account healthcare utilization. Additional studies on health workforce financing inequality using the demand-side approach such as in the utilization or benefit impact analysis of HRH financing could complement this study. Lastly, this study only addresses one dimension of equity which is in relation to geographical distribution. Other dimensions of equity could be assessed such as economic status and ethnicity.

The findings from this study can be useful for other countries with ongoing reform towards universal health coverage [24]. Experience from Thailand showed that improving healthcare coverage could lead to more equitable health workforce financing if the accompanying financial reform be cautious about hospital payment mechanisms that would be responsive to needs. Evidence from other lower and middle-income countries such as Chile, Colombia, Zambia and Zimbabwe also support that more equitable distribution across provinces could be achieved if appropriate resource allocation formulae were used [13].

## Conclusions

Experience from Thailand demonstrates that health workforce financing is an important area that should be considered as part of the overall health system governance. Rising healthcare costs is a major concern in many countries, and a significant proportion is coming from payments to health workers. With the complexity of human resource for health management system, it may not be easy to advocate for effective changes towards a more efficient and equitable health workforce system. The analysis of health workforce financing distribution is one possible tool to help identify potential areas of improvement to ensure that the health workforce system is progressing. Policy makers and academic researchers should pay more attention to the distribution of health workforce financing in their health system planning and strategy development. Our analysis of equalities in health workforce spending shows an improving trend in equity across provinces, and expansion of healthcare and health insurance coverage coupled with financing reform towards demand-side financing had helped to improve equity in the distribution of funding for the health workforce in Thailand.

## References

[1] Kanchanachitra C, Lindelow M, Johnston T, Hanvoravongchai P, Lorenzo FM, Huong NL (2011). Human resources for health in southeast Asia: shortages, distributional challenges, and international trade in health services. Lancet..

[2] Hanvoravongchai P, Ruangrattanatrai W (2012). A review report situation analysis of producing developing and managing HRH system by the statute of health personnel.

[3] Carr-Hill R, Chalmers-Dixon P (2005). The public health observatory handbook of health inequalities measurement.

[4] Ehrenberg RG, Smith RS (1999). Modern labor economics theory and public policy.

[5] Robin B, Michael P (2003). Foundations of microeconomics.

[6] Todaro MP, Smith SC (2003). Economic Development.

[7] Speybroeck N, Ebener S, Sousa A, Paraje G, Evans D, Prasad A (2006). Inequality in access to human resources for health: measurement issues.

[8] De Maio FG (2007). Income inequality measures. J Epidemiol Community Health.

[9] Gupta N, Zurn P, Diallo K, Dal Poz MR (2003). Uses of population census data for monitoring geographical imbalance in the health workforce: snapshots from three developing countries. Int J Equity Health..

[10] Munga MA, Maestad O (2009). Measuring inequalities in the distribution of health workers: the case of Tanzania. Hum Resour Health..

[11] Ono K, Visonnavong V, Konyama K, Hiratsuka Y, Murakami A (2009). Geographical distribution of eye health professionals and cataract surgery in Lao People’s Democratic Republic. Ophthalmic Epidemiol..

[12] Tao Y (2014). Methods for measuring horizontal equity in health resource allocation: a comparative study. Heal Econ Rev..

[13] Anselmi L, Lagarde M, Hanson K (2015). Equity in the allocation of public sector financial resources in low- and middle-income countries: a systematic literature review. Healt781h Policy Plan.

[14] NaRanong V, NaRanong A (2007). Indicators of the health equity No.2.

[15] Srithamrongsawats S, Pannarunothai S (2000). Benchmarks of fairness for evaluating the Thai health care reforms.

[16] Ministry of Public Health, Annual Report of Health Care Resources, available at [http://bps.ops.moph.go.th/Healthinformation/hos55/hos.html]

[17] Hanvoravongchai P (2013). Health financing reform in Thailand: toward universal coverage under fiscal constraints, The World Bank’s Universal Health Coverage Studies Series.

[18] Gastwirth JL (1972). The estimation of the Lorenz curve and Gini index. Rev Econ Stat.

[19] O’Donnell O et al. The concentration index. Chapter 8 in analyzing health equity using household survey data a guide to techniques and their implementation. Washington DC: The World Bank; 2008

[20] StataCorp. Stata statistical software: release 12. , TX: StataCorp LP. Texas: a Stata Press Publication StataCorp LP College Station; 2011.

[21] Ministry of Public Health (2010). Human resources for health: country profile Thailand.

[22] Hernandez-Peña P (2013). Health worker remuneration in WHO Member States. Bull World Health Organ..

[23] National Health Security Office (2012). Thailand’s Universal Coverage Scheme: achievements and challenges An independent assessment of the first 10 years (2001–2010).

[24] Minh HV (2014). Progress toward universal health coverage in ASEAN. Global Health Action..

